# Dementia patients have greater anti-cholinergic drug burden on discharge from hospital: A multicentre cross-sectional study

**DOI:** 10.1192/j.eurpsy.2021.1127

**Published:** 2021-08-13

**Authors:** J. Randall, A. Hook, C-M. Grubb, N. Ellis, J. Wellington, A. Hemmad, A. Zerdelis, B. Geers, B. Sykes, C. Auty, C. Vinchenzo, C. Thorburn, D. Asogbon, E. Granger, H. Boagey, J. Raphael, K. Patel, K. Bhargava, M.-K. Dolley, M. Maden, M. Shah, Q. Lee, R. Vaidya, S. Sehdev, S. Barai, S. Roche, U. Khalid, J. Harrison, D. Codling

**Affiliations:** 1 School Of Medicine, Cardiff University, Cardiff, United Kingdom; 2 Medical School, Newcastle University, Newcastle, United Kingdom; 3 James Cook University Hospital, Middlesbrough, United Kingdom; 4 School Of Medicine, University of Manchester, Manchester, United Kingdom; 5 Medical School, University of Exeter, Exeter, United Kingdom; 6 Lancaster Medical School, Faculty Of Health And Medicine, Lancaster University, Lancaster, United Kingdom; 7 Medical School, University of Bristol, Bristol, United Kingdom; 8 Birmingham Medical School, College Of Medical And Dental Sciences, University of Birmingham, Birmingham, United Kingdom; 9 Acute Medicine, University Hospitals of Morecambe Bay NHS Foundation Trust, Kendal, United Kingdom; 10 Medical Sciences Division, University of Oxford, Oxford, United Kingdom; 11 Norwich Medical School, University of East Anglia, Norwich, United Kingdom; 12 Peninsula Medical School, The Faculty Of Medicine And Dentistry, Plymouth University, Plymouth, United Kingdom; 13 Medical School, Bart’s and The London School of Medicine and Dentistry, London, United Kingdom; 14 Faculty Of Medicine, University of Southampton, Southamptom, United Kingdom; 15 School Of Clinical Medicine, University of Cambridge, Cambridge, United Kingdom; 16 Medical School, University College London, London, United Kingdom; 17 Cardiff University Brain Research Imaging Centre (cubric), Cardiff University, Cardiff, United Kingdom; 18 Institute Of Psychiatry, King’s College London, London, United Kingdom

**Keywords:** dementia, Cholinesterase Inhibitors, Alzheimer Disease, Muscarinic Antagonists

## Abstract

**Introduction:**

Anticholinergic medications block cholinergic transmission. The central effects of anticholinergic drugs can be particularly marked in patients with dementia. Furthermore, anticholinergics antagonise the effects of cholinesterase inhibitors, the main dementia treatment.

**Objectives:**

This study aimed to assess anticholinergic drug prescribing among dementia patients before and after admission to UK acute hospitals.

**Methods:**

352 patients with dementia were included from 17 hospitals in the UK. All were admitted to surgical, medical or Care of the Elderly wards in 2019. Information about patients’ prescriptions were recorded on a standardised form. An evidence-based online calculator was used to calculate the anticholinergic drug burden of each patient. The correlation between two subgroups upon admission and discharge was tested with Spearman’s Rank Correlation.

**Results:**

Table 1 shows patient demographics. On admission, 37.8% of patients had an anticholinergic burden score ≥1 and 5.68% ≥3. At discharge, 43.2% of patients had an anticholinergic burden score ≥1 and 9.1% ≥3. The increase was statistically significant (rho 0.688; p=2.2x10^-16^). The most common group of anticholinergic medications prescribed at discharge were psychotropics (see Figure 1). Among patients prescribed cholinesterase inhibitors, 44.9% were also taking anticholinergic medications.

**Figure:**
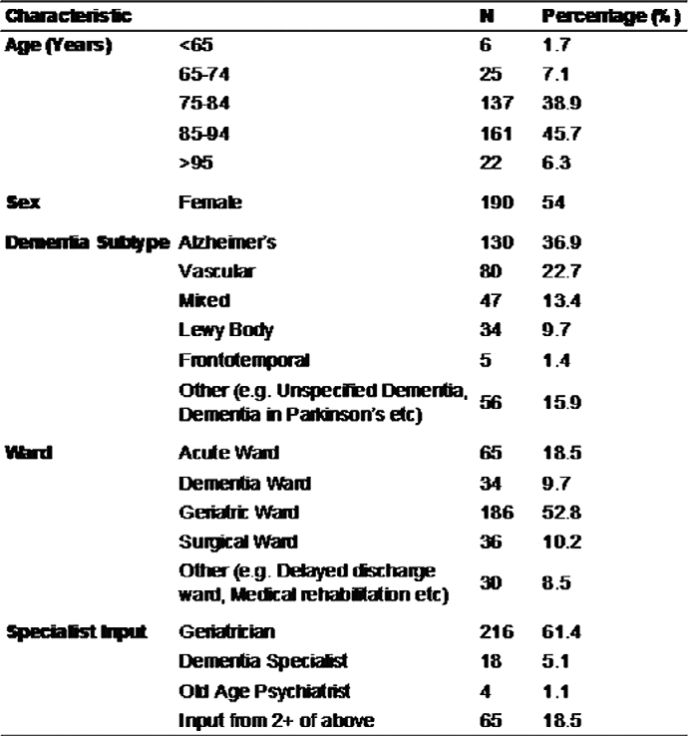

**Figure:**
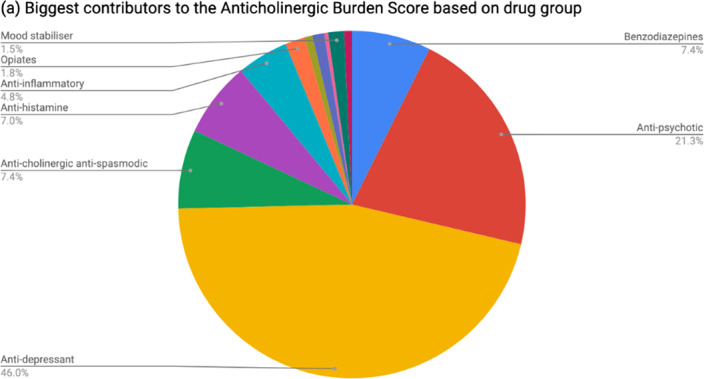

**Conclusions:**

This multicentre cross-sectional study found that people with dementia are frequently prescribed anticholinergic drugs, even if also taking cholinesterase inhibitors, and are significantly more likely to be discharged with a higher anticholinergic drug burden than on admission to hospital.

**Conflict of interest:**

This project was planned and executed by the authors on behalf of SPARC (Student Psychiatry Audit and Research Collaborative). We thank the National Student Association of Medical Research for allowing us use of the Enketo platform. Judith Harrison was su

